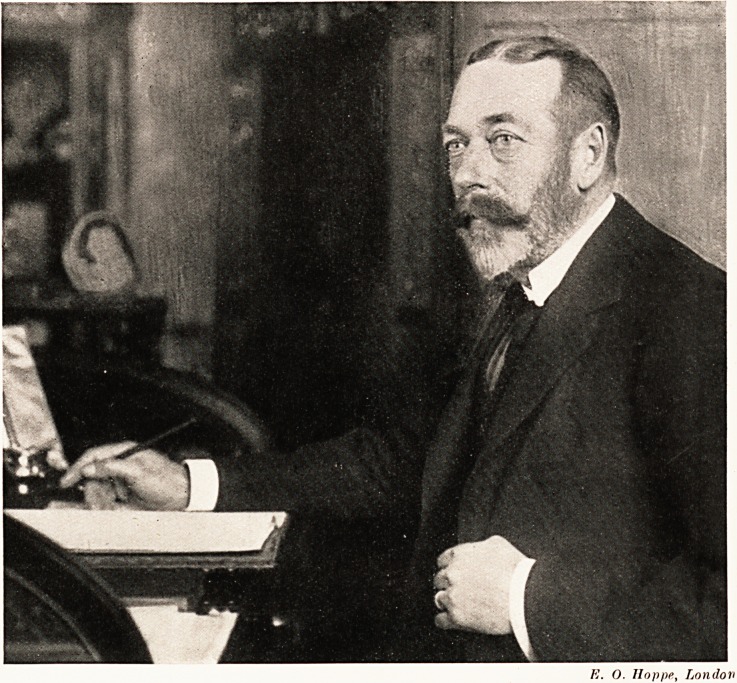# King George V

**Published:** 1936

**Authors:** 


*E. O. Hoppe, London*

### In Memoriam

### KING GEORGE V.

ON Monday night, January 20th, the life of King George V drew peacefully to its close. It would be impossible to write of him in more fitting words than those used by the Prime Minister, Mr. Baldwin, in the House of Commons:—

It was in the reign of King George V that the greatest and swiftest changes occurred; and he met the challenge of the times without flinching, and he triumphed at a time when a slip of speech even, or of action, might have wrought irreparable damage. Day by day he discharged those duties which thronged upon him, with his will rigorously trained to place the public interest first and last. His own ease and pleasure were never considered. I cannot tell you how it happened, as you all know it did, that the sure instinct of our people gradually discerned that whatever human frailties or limitations might have attached to their King, his sense of duty to his people amounted to genius. He communicated his personality by some indefinable, intangible wave of sympathy and understanding to every one of his subjects, not only at home but throughout the world.

He was taken away peacefully. He fell asleep with no pain, no suffering, no apprehensions, at peace with all the world, and it was not given to him to have that last trial that I think he would have found more difficult to bear than any man I know—having to continue his work with a failing body, or possibly with a failing mind. He was taken away from us, delicate it is true, feeling the effects of that last illness, it is true, but with little loss of physical and no loss of mental powers. Those of us whose duty it was to see him frequently have no memory of him but at his best, and his best was something very fine.

## Figures and Tables

**Figure f1:**